# Henoch Schönlein purpura associated with pulmonary adenocarcinoma

**DOI:** 10.1186/1752-1947-5-226

**Published:** 2011-06-22

**Authors:** Daiki Mifune, Satoshi Watanabe, Rie Kondo, Yoko Wada, Hiroshi Moriyama, Hiroshi Kagamu, Hirohisa Yoshizawa, Takafumi Tetsuka, Asako Matsuyama, Kaoru Ito, Ichiei Narita

**Affiliations:** 1 Department of Medicine (II), Niigata University Medical and Dental Hospital, 951-8520 Niigata, Japan; 2 Bioscience Medical Research Center, Niigata University Medical and Dental Hospital, 951-8520 Niigata, Japan; 3 Division of Respiratory Medicine, Niigata City General Hospital, 950-1197 Niigata, Japan; 4 Division of Dermatology, Niigata University Graduate School of Medical and Dental Sciences, 951-8510 Niigata, Japan

## Abstract

**Introduction:**

Henoch-Schönlein purpura is a common immunoglobulin A-mediated vasculitis syndrome in children. Henoch-Schönlein purpura can also affect adults and is probably related to malignancy.

**Case presentation:**

We report the case of a 61-year-old Japanese man who presented for examination after an abnormal shadow was detected by chest radiography. He received a diagnosis of pulmonary adenocarcinoma, stage IV. Purpura on the legs, abdominal pain, diarrhea, hematuria and proteinuria developed at this time. Henoch-Schönlein purpura was diagnosed, base on the clinical symptoms and histological findings of biopsy specimens of the skin, which showed vasculitis with immunoglobulin A deposits. Our patient received chemotherapy with gemcitabine after successful steroid therapy for the Henoch-Schönlein purpura.

**Conclusion:**

Although hematological malignancies are well-known causes of vasculitides, cases of Henoch-Schönlein purpura associated with lung adenocarcinoma are rare. Our patient was treated with corticosteroid therapy, which cleared the purpura and cytotoxic chemotherapy for the non-small cell lung cancer. However, he died from heart failure due to cardiac tamponade.

## Introduction

Henoch-Schönlein Purpura (HSP) is a systemic vasculitis that involves the small vessels, most notably those in the skin, gastrointestinal tract and glomeruli, and is accompanied by arthralgia or arthritis [1]. HSP occurs commonly in children but can also affect adults [2]. Although the exact cause of HSP remains unknown, malignancy has been reported as a causative factor [3-5]. This report illustrates a rare case of HSP associated with pulmonary adenocarcinoma.

## Case Presentation

**Figure 1:**
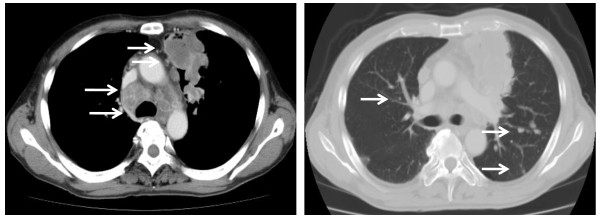
**Computed tomography (CT) scan of the chest on admission to our hospital**. Tumor in the left upper lobe, swelling of mediastinal lymph nodes, and small nodules in both lung fields and pleura (arrows).

A 61-year-old Japanese man presented for examination after an abnormal lung shadow was detected during population-based radiological screening. He was a current smoker with a 41 pack-year smoking history. Chest computed tomography (CT) showed a mass measuring 60 × 50 mm in the left upper lobe, with small nodules in both lung fields and left pleura, and mediastinal and subclavicular lymphadenopathy (Figure 1). Bronchoscopy revealed stenosis of the left B3 bronchus with mucosal abnormalities at points from which biopsy specimens were obtained. Cerebral magnetic resonance imaging (MRI) demonstrated multiple brain metastases. The final oncological diagnosis was a stage IV adenocarcinoma (T4N3M1b) without epidermal growth factor receptor gene mutation. After stereotactic radiosurgery for brain metastases, carried out in another hospital, our patient was referred to us to receive chemotherapy.

**Figure 2:**
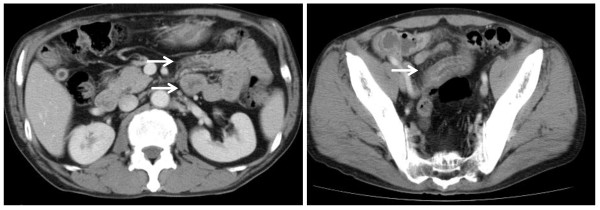
**Computed tomography (CT) scan of the abdomen and pelvis on admission to our hospital**. No evidence of metastasis but did identify diffuse swelling and thickening of the small intestine walls (arrows).

**Figure 3:**
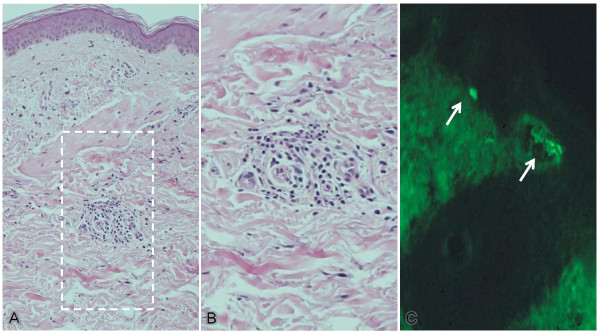
**Histopathology of the palpable purpura**. **(A) **Cellular infiltration into the obscured small vessel walls of the subpapillar plexus under the epidermis (hematoxylin and eosin, original magnification × 100). **(B) **Infiltration of polymorphonuclear cells (haematoxylin and eosin, original magnification × 150) **(C) **Fluorescent immunoglobulin A deposits (arrows) (original magnification × 100).

On admission, our patient had progressive abdominal pain, diarrhea and purpuric rash on the legs, which had developed over the previous month. He was not taking any medication. The abdomen was soft and flat with minor tenderness. Palpable purpura was present on both legs. His Eastern Cooperative Oncology Group performance status was 1. His hematologic and blood chemical values were: white blood cell count of 9530/mm^3^, a hemoglobin concentration of 11.2 g/dL, an erythrocyte sedimentation rate of 83 mm/hour, 6.52 mg/dL serum C-reactive protein, >55U/mL CH50, 132 mg/dL C3, 37.7 mg/dL C4, 10.7 μg/dL D-dimer and 694 mg/dL fibrinogen (normal 160-400). Urine analysis showed proteinuria of 1.28 g/day and hematuria of >100 erythrocytes per high-power field. Stool examination for occult blood was positive. The findings were normal or negative for platelet count, prothrombin time, activated partial thromboplastin time, transaminases, creatinine, antistreptolysin O, rheumatoid factor (RF), antinuclear antibody (ANA), IgA, antineutrophil cytoplasmic antibody (ANCA), cryoglobulin, rapid plasma regain, hepatitis B surface antigen (HBsAg), hepatitis C virus (HCV) antibody and human immunodeficiency virus antibody. Blood, urine and sputum cultures were negative. Abdominal CT revealed diffuse swelling and thickening of the small intestine walls and an absence of metastases (Figure 2). Enteroscopy demonstrated multiple erosions and ulcers of the intestine. A biopsy taken from the intestinal mucus showed non-specific chronic inflammation without evidence of vasculitis. On histopathologic examination of the palpable purpura, vasculitis was seen, with infiltration of polymorphonuclear cells into the vessel walls of the subpapillar plexus under the epidermis (Figure 3AB). Immunofluorescent studies of the biopsy specimen revealed deposition of IgA without C3 in superficial blood vessels (Figure 3C).

Our patient was diagnosed with HSP. Treatment with prednisolone 0.8 mg/kg/day was initiated following deterioration of renal function, as evidenced by a drop in creatinine clearance from 82 mL/min at the time of admission to 27 mL/min before starting prednisolone. The clinical features of HSP, including abdominal pain and skin rash, resolved within a week. Creatinine clearance improved to 46 mL/min four weeks after the start of steroid treatment. Prednisolone was tapered and maintained at 0.6 mg/kg/day. Our patient received two cycles of chemotherapy with gemcitabine 1000 mg/m^2 ^administered on days one and eight of each three-weeks cycle. HSP was well controlled, and no other adverse events were observed during chemotherapy. Two months after of the start of chemotherapy, the patient developed rapidly progressing dyspnea and hypoxia. Chest CT and cardiac ultrasonography identified tumor invasion of the pericardium and massive pericardial effusion. Cytological examination of the pericardial effusion revealed adenocarcinoma cells. Our patient was diagnosed with heart failure due to cardiac tamponade, and twice underwent surgical drainage of the pericardial effusion. His general condition gradually deteriorated with disease progression, and he died three months after the onset of HSP.

## Discussion

HSP is a systemic vasculitis with IgA-dominant immune deposits affecting small vessels, especially those in the skin, gastrointestinal tract and kidney, and is accompanied by arthralgia or arthritis [1]. HSP occurs more commonly in children. The clinical presentation of HSP is more severe in adults than in children, and the outcome in adults is relatively poor [2].

The diagnostic criteria for HSP by the American College of Rheumatology are: palpable purpura, patient aged ≤20 years; bowel angina, and granulocytes in the walls of small arterioles or venules seen on biopsy. The presence of two or more of these criteria has a sensitivity and specificity of 87.1% and 87.7%, respectively [6]. Our case met three of the four criteria, with the exception of age. Other vasculitides were highly unlikely because markers for other systemic vasculitides (antistreptolysin O, RF, ANA, ANCA, cryoglobulin, rapid plasma regain, HBsAg, HCV) were all negative. Moreover, immunofluorescent studies of the biopsied skin specimen showed IgA deposition in blood vessels. These findings confirmed HSP. Novák *et al*. reported that vasculitic endoscopic lesions had been documented in only some patients with HSP with abdominal symptoms [7]. Their findings may explain the finding in our patient's intestinal specimen of non-specific chronic inflammation without vasculitis.

The occurrence of vasculitis in patients with malignancy has been estimated at one in 1800 for hematological malignancies and one in 80,800 for solid tumors [3]. HSP represents only 5% of vasculitis cases associated with malignancy [4]. Although hematological malignancies were more common than solid tumors in all types of vasculitis, solid tumors were more common in association with HSP [5]. Mitsui *et al*. reported that 23 of 53 patients with HSP (43.4%) aged >40 years had underlying malignancies [8]. The etiology of HSP remains obscure, and various triggers, including bacterial or viral infections, foods and drugs, have been hypothesized. Adult patients with HSP should be investigated for malignancies, especially in the absence of these triggers [8].

Lung cancer is the most common solid malignancy associated with HSP. Twelve cases of lung cancer presenting with HSP have been reported to date, including this one (Table 1) [9-18]. Mean patient age was 64.9 years (range 50-79 years) and all patients were men. The most common lung-cancer histological diagnoses were squamous cell (n = 7), adenocarcinoma (n = 3) and small cell (n = 2). In six cases, the diagnosis of both processes was simultaneous, and in four cases, HSP antedated lung cancer by an average of 9.2 months (range five-22 months). Lung cancer did not antedate HSP in any of the cases. All patients had palpable purpura and renal involvement, 55% of patients had gastrointestinal symptoms and 64% had joint involvement. In the case reported here, there were no identifiable triggers for the onset of HSP other than the malignancy itself. The diagnosis of malignancy and the onset of HSP occurred at the same time, therefore we believe that the HSP was associated with the malignancy.

**Table 1:** Patients with Henoch-Schönlein purpura and lung cancer.

Author	Histology	Occurrence of vasculitis in relation to tumor	HSP therapy	Malignancy therapy
Cairns [9]	Squamous	eight months before tumor	Not done	None
	Squamous	Synchronous^a^	Not done	Surgical resection
Maurice [10]	Squamous	Synchronous	Not done	Surgical resection
Mitchell [11]	Squamous	22 months before tumor	Prednisone + azathioprine	Surgical resection
Pfitzenmeyer [12]	Squamous	six months before tumor	Not done	None
Gutiérrez [13]	Squamous	Synchronous	Not available	Not available
Frigui [14]	Squamous	six months before tumor	Methylprednisolone pulses, prednisone	Chemotherapy, radiotherapy
Blanco R [15]	Small cell	Synchronous	Not done	Chemotherapy
Ponge T [16]	Small cell	eight months before tumor	Not done	Chemotherapy
Weiler-Bisig [17]	Adenocarcinoma	five months before tumor	Prednisolone	Chemotherapy
Salon [18]	Adenocarcinoma	Synchronous	Prednisone + intravenous gammaglobulins	Surgical resection
Present case	Adenocarcinoma	Synchronous	Prednisolone	Chemotherapy

^a^Synchronous: within 1 month of cancer diagnosis.

Treatment for patients with HSP and lung cancer is also summarized in Table 1. Therapies for lung cancer induced remission of HSP in some cases [9,10,15,16], whereas other case reports indicated that corticosteroid therapy was required for improvement of HSP [11,14,17,18], as with our case. Our patient had an Eastern Cooperative Oncology Group performance status of 1, and after treatment of the HSP to correct the progressive renal insufficiency, there was no contraindication for chemotherapy. Corticosteroid therapy obviously contributed to the improvement of the HSP and safe chemotherapy. Unfortunately, the patient did not respond to chemotherapy and died from causes associated with the underlying cancer. Mitsui *et al*. found a correlation between the development of HSP and poor prognosis of malignancies [8]. They reported that seven of nine patients with HSP and malignancies exhibited new metastatic lesions or died of cancer within one-32 months from the onset of HSP.

## Conclusion

We report a rare case of HSP associated with non-small cell lung cancer. To the best of our knowledge, this is only the third case of HSP presenting with pulmonary adenocarcinoma. Although the optimum treatment of HSP with malignancy remains unclear, corticosteroid therapy dramatically improved our patient's clinical symptoms, and contributed to the management of his lung cancer.

## Consent

Written informed consent was obtained from the patient's spouse for publication of this case report and any accompanying images. A copy of the written consent is available for review by the Editor-in-Chief of this journal.

## Competing interests

The authors declare that they have no competing interests.

## Authors' contributions

DM wrote the manuscript. SW was responsible for the manuscript concept and final corrections to the manuscript. YW and IN analyzed and interpreted our patient data regarding the renal disease. AM and KI performed the histological examination of the skin. RK, HM, HK, HY and TT participated in patient care and collected data. All authors have read and approved the final manuscript.
